# Factor structure and internal consistency of the parent patient activation measure (P-PAM) in parents of children with ADHD in norwegian paediatric mental health

**DOI:** 10.1186/s12888-023-04550-0

**Published:** 2023-01-23

**Authors:** Ingunn Mundal, Petter Laake, Stål K. Bjørkly, Mariela L. Lara-Cabrera

**Affiliations:** 1 Faculty of Health Siences and Social care, Molde University College, Molde, Norway; 2 Division of Psychiatry, Kristiansund Community Mental Health Centre, Møre Og Romsdal Hospital Trust, Kristiansund, Norway; 3 Regional Centre for Child and Youth Mental Health and Child Welfare (RKBU), Department of Mental Health, Faculty of Medicine and Health Sciences, Norwegian University of Science and Technology (NTNU), Trondheim, Norway; 4 Department of Biostatistics, Oslo Centre for Statistics and Epidemiology, University of Oslo, Oslo, Norway; 5 Oslo University Hospital, Centre for Forensic Research, Oslo, Norway; 6 Department of Mental Health, Faculty of Medicine and Health Sciences, Norwegian University of Science and Technology (NTNU), Trondheim, Norway; 7 Division of Psychiatry, Nidelv Community Mental Health Centre, St. Olav’s University Hospital, Trondheim, Norway

**Keywords:** Paediatrics, Parent engagement, Attention-deficit/hyperactivity disorder, Confirmatory factor analysis, Internal consistency, Patient Activation Measure

## Abstract

**Background:**

This study aimed to explore the internal consistency and factor validity of the 13-item self-report questionnaire Parent-Patient Activation Measure (P-PAM) in a sample of parents of children with Attention-deficit/hyperactivity disorder.

**Methods:**

In a cross-sectional study, 239 parents were recruited from four outpatient clinics of the Child and Adolescent Mental Health Services and completed the P-PAM along with demographic variables. The factor structure of the P-PAM was examined through exploratory factor analysis, and internal consistency was estimated with the use of both Cronbach’s alpha and McDonald’s omega. A confirmatory factor analysis was used to estimate and test individual parameters.

**Results:**

The fit indices suggest an acceptable two-factor model of P-PAM and show high internal consistency and reliability for both factors, indicating that the scale measures two concepts.

**Conclusions:**

Our findings provide evidence for an acceptable factor structure and a high reliability of P-PAM as a measure of parent activation, suggesting that the theoretical factors reflect the construct of parent activation as intuitively compiled into an inner cognitive factor and an outer behavioral factor, which are related.

## Background

Parent activation, or parent healthcare activation on behalf of their child, involves the parents’ willingness to take independent actions to manage their child’s behavioural healthcare in different aspects of engagement [1, 2]. Healthcare activation is defined as the knowledge, confidence, willingness and skills to manage one’s health and healthcare [1, 3, 4]. High levels of activation has been associated with improved health and healthcare outcomes in adult populations [5–9]. Parent activation, on the other hand, is regarded as an essential component of disease management in terms of their responsibility to ensure adherence to treatments, find the way through and make decisions about help-seeking to address their child’s problems [3]. However, in paediatric contexts there are still limited data related to factors that contribute to parent activation [2].

In ill paediatric populations, for example in Child and Adolescents Mental Health Services (CAMHS), most parents adopt a significant role in the co-management of their child’s condition [10]. Conditions such as the highly prevalent Attention-deficit/hyperactivity disorder (ADHD) have a significant impact on both the child and their family [11], and parents of children with ADHD have to cope with demanding regimens, stressful treatments, issues related to adherence to treatment and an extended recovery period. Therefore, parent participation in care is critical [12], and the level of their engagement and involvement may influence their child´s treatment and thus recovery or management of the condition positively.

The 13-item Parent-Patient Activation Measure (P-PAM) and the concept of parent activation have been theorized and adapted from the 13-item self-report Patient Activation Measure (PAM-13) [13] and was designed for use among caregivers of paediatric patients [12]. The questionnaire aims to include a quantitative measure of all aspects of parent engagement on behalf of their child [14] and also represents the individual level of engagement [2]. The initial assessment of the P-PAM has shown a high internal consistency value (Cronbach’s alpha for PAM-13, α = 0.86; and P-PAM α = 0.85) and a high correlation to the PAM-13 in terms of the univariate comparison of PAM-13 and P-PAM (*r* = 0.55, *p* < 0.001) [12]. However, there have been limited empirical studies to validate the internal consistency and factor structure to implement the P-PAM for use in clinical settings [12, 14–16]. Even though the original PAM-13 has shown good psychometric properties across various contexts [3, 7, 12, 14, 17–22], there is a scarcity in research results that have confirmed that the factor model of the P-PAM is consistent with the theory of the PAM-13 and the four-factor model (beliefs, confidence, action, and perseverance) for parent activation [4, 14–16].

To model the PAM-13, the inventors used Rasch analysis to allow the measurement to produce interval level variables and to provide a unified approach to test the validity in different patient populations [13]. However, exploratory or confirmatory factor analysis has frequently been used to assess the dimensionality of the items, namely which items measure which factors if the item set is multidimensional, and to examine which items fail to load on any factor [15].

It is important to understand what factors influence parent activation, and how to target these factors to develop and deliver more helpful interventions [23]. Moreover, to ensure the appropriateness of the P-PAM, it is important to validate the questionnaire for its intended population, as the prevalence and informant perception of mental health problems may vary across cultures and groups [24]. Thus, further research is vital to explore the relevance of the P-PAM in parents of children with ADHD, and to assess the psychometric properties of the P-PAM among parents on behalf of their child. The aim of this study was to explore the internal consistency, and factor structure of the self-report questionnaire P-PAM in a sample of parents of children with ADHD in the CAMHS in Norway.

## Methods

### Study design

We conducted a cross-sectional study to examine the psychometrics of the Norwegian version of the P-PAM questionnaire, with the participants of parents in four outpatient clinics in the CAMHS in Norway. The P-PAM was used to evaluate the healthcare activation in parents on behalf of their child with any type of ADHD. Participation was anonymous and voluntary, and only parents of children diagnosed with ADHD were included in this study.

### Participants and procedure

Norwegian-speaking parents (*n* = 239) of children with ADHD were recruited, and almost 90% of the caregivers were biological parents. The 13-item P-PAM, together with a few demographic questions were included in the data collection and scored on paper and pencil questionnaires.

Parents of children newly diagnosed with ADHD were recruited between May 2019 and February 2020, in the context of their attendance at a one-day course specifically designed for parental education on ADHD, which they had signed up for in advance.

The individual child’s therapist completed all the routine assessments and diagnostic processes and coding according to the Diagnostic and Statistical Manual of Mental Disorders version IV (DSM-IV), International Statistical Classification of Disease and Related Health Problems (ICD-10) [25], and the Norwegian national guideline for ADHD, comprising information from patients, parents and teachers; developmental history; somatic health status; and school functioning [26, 27]. ADHD symptoms were assessed with the Achenbach system of empirically based assessment (ASEBA) checklists [28] and the ADHD Rating Scale-IV (ADHD-RS-IV) [29]. Adaptive functioning and IQ scores were obtained using the Children’s Global Assessment Scale (CGAS) [30] and Wechsler Intelligence Scales for Children [31]. After the child has been diagnosed with ADHD, the therapist invited the parents to participate in an educational group intervention.

Ahead of the parental course, the questionnaires were distributed to the parents and provided a concise information regarding the project. Parents who were interested in participating received an anonymous envelope with written information and the questionnaires, which they were asked to complete by the end of the course. Consent implied that the parents received both oral and written information, and it was implicitly given by anonymously responding to the questionnaires and returning the envelope at the end of the course. The study was approved by the Regional Committee for Medicine and Health Research Ethics in Mid-Norway (ref.: 2018/1196).

### Measurements

#### Parent-Patient Activation Measure (P-PAM)

Parent activation was assessed using the self-rated 13-item P-PAM, which require parents to assess their knowledge, confidence and willingness to act in regard to their child’s health [12]. The P-PAM is licenced by Insignia Health and was used with their permission [32]. Response options were on a four-point Likert scale ranging from (1) ‘disagree strongly’ to (4) ‘agree strongly’ or the option of (5) ‘not applicable’. The total activation score was calculated on a continuous scale ranging from 0–100 and stratified into four levels of activation with higher scores corresponding to higher activation [6, 14, 15]. At Level 1 = 0–47.0, parents may not yet understand that their own role is important; at Level 2 = 47.1–55.1, parents lack the confidence and knowledge to take action; at Level 3 = 55.2–72.4, parents engage in recommended health behaviours in support of their child; and at Level 4 = 72.5–100, parents are actively managing their child’s health and engaging in recommended health behaviours.

### Statistical analysis

The statistical procedures were performed in Stata statistical software version 16.0 [33] and SPSS version 28.0.1 with an added OMEGA macro. Descriptive statistics were presented for demographic variables and included means, standard deviations (SD), and percentages.

We first conducted an exploratory factor analysis (EFA) to investigate the factor structure in P-PAM, to explore the group validity to consider the numbers of latent factors and to summarize interpretation and dimensionality. To ensure sampling adequacy and that the EFA is appropriate, the Kaiser-Maier-Olkin (KMO) test of sampling adequacy above 0.5 and Bartlett’s test of sphericity with a significant *p*-value < 0.001 were used to confirm a correlation among the included variables and to determine whether the data were fitting a factor analysis. A one-component and an alternative two-component solution were compared by extracting components with eigenvalues greater than 1 as well as exploring the scree plots, and by using an oblique Oblimin rotation, assuming the factors being correlated [34].

A confirmatory factor analysis (CFA) was used to estimate and test individual parameters and to inspect the interrelations among the latent construct and observable variables [35]. The prespecified factor structure for parent activation suggested by the EFA and confirmed in CFA was evaluated with respect to goodness of fit. Score reliability was estimated from the CFA, using both Cronbach’s alpha (*α*) [36] and McDonald’s omega (ω) [37], expecting coefficients above 0.70 as a threshold for both coefficients [38]. Both approaches are estimates of internal consistency; however, the infrequently reported McDonald’s ω may be regarded as a better option as it is less restrictive than Cronbach’s α in allowing the means and variances of true scores, as well as the error variance, to vary [24, 39]. Furthermore, the assumption of tau-equivalence (i.e. the same true score for all test items, or equal factor loadings) is a requirement of the Cronbach’s α, which is not the case with McDonald’s ω [37, 40]. A *p*-value ≤ 0.05 was considered significant. Parameters for fit estimation were the χ2 (chi-square), the CFI (comparative fit index), the TLI (Tucker Lewis index), the RMSEA (the root mean square residual), and the SRMR (the standardized root mean square residual) [35]. Because of the “not applicable” response option in P-PAM, only complete questionnaires were included in the analyses. Due to missing P-PAM scores for 96 respondents, statistical differences between complete and incomplete questionnaires were explored using two-sample *t*-test for continuous variables (age for parents and child) and Pearson’s chi-square test for categorical variables (gender for parents and child, and education).

## Results

Of the 239 parents who completed the P-PAM, 63.1% were mothers and 36.9% were fathers. The mean age of the parents was 40.2 years (*SD* = 7.96), ranging from 26 to 71 years. The demographic characteristics of the participants are shown in Table 1. There were no statistically significant differences between those with complete P-PAM responses (*n* = 143) and those with missing P-PAM responses (*n* = 96). Table 2 shows the descriptive scores of the items in the P-PAM. According to the total scores for the P-PAM, the overall sample mean was 76.2 (*SD* = 15.81). Most of the parents (68.5%, *n* = 98)) scored at the highest activation level 4, and 25.2% (*n* = 36) scored at level 3, indicating that only 6.3% (*n* = 9) at the lowest level 1 and 2.

**Table 1:** Sample characteristics of parents of children with ADHD

	**Parents ** **(*n total* = 239)**	**Parents ** **(*n complete cases for P-PAM* = 143)**
**Age Parent (SD)**	40.2 (7.96)	40.5 (8.18)
**Parent**		
Mother **(%)**	149 (63.1)	93 (65.5)
Father	87 (36.9)	49 (34.5)
**Gender child**		
Girl **(%)**	75 (32.8)	44 (31.2)
Boy	154 (67.3)	94 (68.1)
**Age Child (SD**)	10.5 (3.1)	10.7 (3.3)
**Marital status;** (%)		
Single	48 (21.4)	33 (23.9)
Married/cohabitant	176 (78.6)	105 (76.1)
**Education in years;** (%)		
Primary school	14 (5.9)	9 (6.3)
High school	118 (50.0)	76 (53.5)
College/University ≤ 4 years	104 (44.1)	57 (40.1)
**Working Status**^a^(%)		
Full time work	172 (73.5)	100 (71.4)
Student	3 (1.3)	1 (0.7)
Unemployed	2 (0.9)	7 (5.2)
Part time or Sick leave	56 (24.4)	32 (22.7)
**First language (%)**		
Norwegian/Scandinavian	230 (97.9)	140 (99.3)
Other	5 (2.1)	1 (0.7)
**Living place**^a^** (%)**		
City	88 (45.6)	45 (38.5)
Village	104 (53.9)	71 (60.7)

*Abbreviations: ADHD* attention-deficit/hyperactivity disorder, *n* number of parents, *P-PAM* parent patient activation measure

^a^Because of missing values, N vary, and percentages do not sum to 100

**Table 2:** Descriptive statistics of the P-PAM items (factor loading), *n* = 143

Item	Mean	Standard deviation	Factor loadings Factor 1 Factor 2
1. I am the person who is responsible for taking care of my child	3.86	.51	.71 -.01
2. Taking an active role in my child’s health is the most important thing that affects his/her health	3.83	.48	.86 -.09
3. I am confident I can take actions to help prevent reduce problems associated with my child’s health	3.69	.57	.53 .20
4. I know what each of my child’s prescribed medication do	3.73	.66	.59 .03
5. I am confident that I can tell whether I need to go to the doctor or whether I can take care of my child’s health problem my self	3.58	.62	.40 .39
6. I am confident I can tell a doctor or nurse the concern that I have about my child’s health even when he or she does not ask	3.84	.47	.78 .04
7. I am confident that I can carry out medical treatments I need to do for my child at home	3.83	.49	.72 .06
8. I understand my child’s health problems and what causes them	3.39	.70	.28 .50
9. I know what treatments are available for my child’s health problem	2.96	.80	-.06 .72
10. I have been able to help my child maintain (keep up with) lifestyle changes, like healthy eating or exercising	3.31	.75	.05 .76
11. I know how to prevent problems with my child’s health	3.24	.66	.01 .77
12. I am confident I can work out solutions when new problems arise with my child’s health	3.26	.70	-.04 .77
13. I am confident that I can help my child maintain lifestyle changes, like healthy eating and exercise, even during times of stress	3.27	.72	.00 .77

### Exploratory factor analysis of P-PAM

**Fig. 1:**
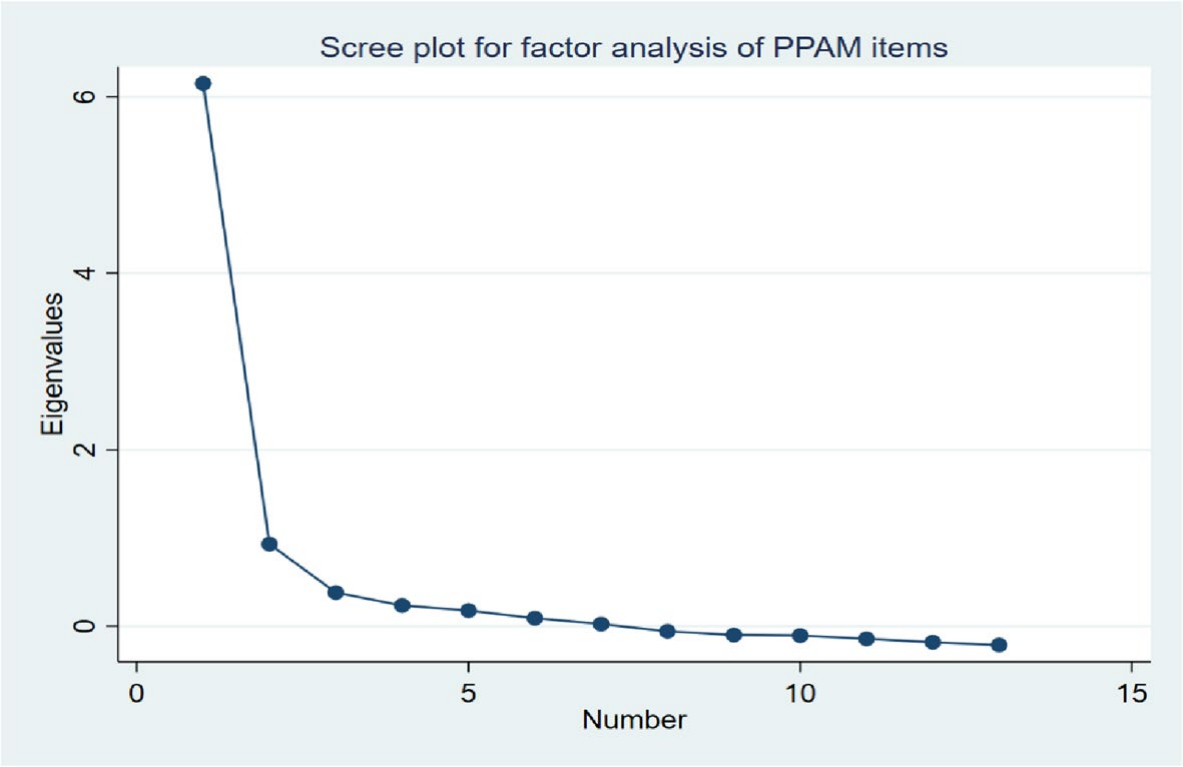
Scree plot of the factor analysis for the P-PAM items

In accordance with the eigenvalue criterion, the analysis suggested a one-factor solution to explain 86% of the variance with an Eigenvalue of 6.15 and varying factor loadings (0.57–0.75). However, the scree plot of the factor analysis supported a two-factor solution for the inflexion point was (see Fig. 1). The EFA with oblique rotation then conducted on the 13 items of the Norwegian P-PAM suggested an extraction of two factors, of which one factor had Eigenvalues that met Kaiser’s criterion of > 1 [41]. However, two-factor solution explained 99% of the variance. The KMO value of sampling adequacy for the items for use in EFA was 0.908 (*p* < 0.001), which indicates a high value, considerably above the recommended values of 0.80 and *p* < 0.05. Bartlett’s test of sphericity (χ2 = 1,018.54, *p* < 0.001) showed significant and sufficiently large correlations between the items for inclusion in the EFA [42]. In addition, in the two-factor model, the first seven items (covering ‘beliefs’, and ‘confidence and knowledge’) were stipulated to identify with the first factor, whereas the last six items (covering ‘action’ and ‘perseverance’) were related to the second factor.

### Inter-item reliability of P-PAM

Table 3 shows the score reliability estimated from the CFA using both Cronbach’s alpha (*α*) and McDonald’s omega (ω). As a result of the two-factor solution following the Oblimin oblique rotation, Factor 1 included items 1–7 (factor loadings differed from 0.40– 0.86) and showed a Cronbach’s α and McDonald’s ω of 0.87. Items 8–13 (factor loadings differed from 0.39 – 0.77) showed a Cronbach’s α and McDonald’s ω of 0.89. Item 5 (I am confident that I can tell whether I need to go to the doctor or whether I can take care of my child’s health problem myself) loaded on both factors with a factor loading of < 0.4. This item was retained because excluding the loadings from each of the item from the analysis did not influence the inter-item reliability.

**Table 3:** Cronbach’s α and McDonalds ω and 95% CI for the parent activation measure. 95% CI and SE are bootstrap estimates

Factor	Cronbach’s α	[95% CI]	SE	McDonalds ω	[95% CI]	SE
Factor 1	.87	[.57 .94]	.94	.87	[.56 .94]	.95
Factor 2	.89	[.84 .92]	.22	.89	[.83 .92]	.22

*Abbreviations: CI* confidence interval, *SE* standard error

### Confirmatory factor analysis of P-PAM

Based on the model of the exploratory factor analysis, we estimated a confirmatory factor model with the aim of testing whether the values of the goodness of fit statistics were acceptable. The chi-square statistics, which are highly sensitive to sample size, were statistically significant (*χ*^2^ = 134,77, *df* = 63, *p* < 0.001); however, the SRMR was 0.06; with the preferable size being < 0.08, and the RMSEA was 0.09, slightly above the preferable size of < 0.08. Regarding the preferable size of 0.93, the CFI and the TLI was 0.93 and 0.91, respectively, suggesting an acceptable two-factor model of the P-PAM.

**Fig. 2:**
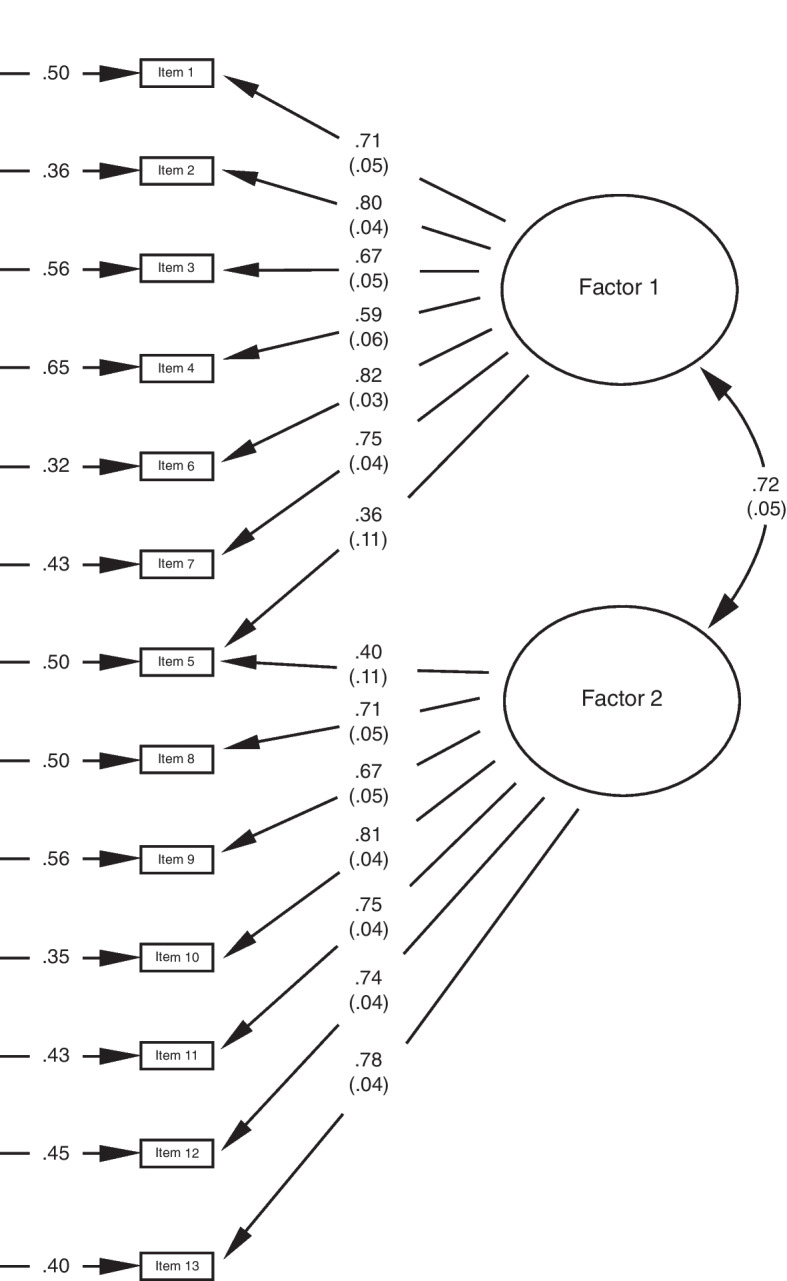
Confirmatory factor analysis for the P-PAM. The loadings are indicated as the number connected from the factors to the items. The unique variances are the numbers connected to the arrows pointing to the items

We used CFA to assess the inter-relations among the factors. Figure 2 shows how the factor structure, the factor loadings and unique variances for each item of the two factors of P-PAM are positively correlated (*r* = 0.72), which imply that the items in the overall concept of parent activation approximately reflect the theoretical factors of ‘beliefs’, and ‘confidence and knowledge’ in Factor 1 and ‘action and perseverance’ in Factor 2. The factor loadings variation between Factor1 1 and 2 presented in Fig. 2, were significant at *p* < 0.05.

## Discussion

Despite contradictory findings in the existing literature on the validation of the P-PAM, the concept of parent activation on behalf of their child is still held as an essential component of disease management in the youth populations. This study aimed to explore the factor structure and estimate the internal consistency of the P-PAM in a sample of parents of children with ADHD in the CAMHS, thus providing important information regarding the P-PAM for use in the CAMHS and mental healthcare research.

### Main findings

#### Factor structure

Our results show that the model fit indices of the CFA suggest that a two-factor model of P-PAM is acceptable and showed high internal consistency and reliability between the factors. The exploratory factor analysis in our study suggests an extraction of two factors and demonstrates a strong positive correlation between the factors. Similar to our findings, factor analyses in previous studies have uncovered that the data did not fit the theoretical four-factor model of parent activation and the theoretical structure of patient activation as four factors (beliefs, confidence, action, perseverance) [14–16]. On the other hand, our findings provide support for the characteristics of the concept of parent activation in that ‘beliefs’ and ‘confidence and knowledge’ represent Factor 1’, and ‘action’ and ‘perseverance’ represent Factor 2.

There are various adapted versions of parent activation measures in circulation, such as the Parent Activation Measure for Developmental Disabilities (PAM-DD) [16], and Parent Patient Activation Measure-Mental Health (P-PAM-MH) [3]. Although evidence indicates that the measures are valid and reliable, it does not necessarily follow that the same applies to different populations and chronic illnesses [13]. In addition, several adapted versions may impede the comparability of studies, as well as increase concerns regarding the underlying factor structure on whether the P-PAM measure the same construct as the PAM. Another aspect is that most studies that have evaluated the P-PAM include and compare English- and Spanish-speaking parents with the aim of evaluating differences in demographics and socio-economic conditions [14–16, 43], which may cause bias in the findings.

#### Internal consistency

An examination of the validity if the P-PAM examined by DeCamp and colleagues provided evidence of good internal consistency [14]. This aligns with our results, which show promising estimates for the internal consistency, exceeding the expected threshold for good levels (0.70) for both *α* and *ω* and reflecting the overall concept of parent activation as well as good internal consistency in the factors. Although most previous studies of P-PAM have reported reliability using Cronbach’s *α,* we included McDonald’s *ω* in our analysis because the methodologists advocate a shift or a disciplinary change with the use of *ω* [37, 44]*.* To explore the psychometric properties of the P-PAM, the reliability evidence of test scores is essential, in that the quality of the use of the P-PAM should be based on a proper interpretation of the test scores [45]. Some of the criticism of the use of Cronbach’s *α* is related to its limited usefulness in calculating scale reliability for multi-item scales because coefficient alpha requires that the data meet a set of specific assumptions that may be ignored, and it is, thus, likely that the reliability of the test scores may be misrepresented [44]. However, in our study, the use of Cronbach’s *α* seems to be an adequate reliability coefficient because it does meet the assumption of tau-equivalence, which is demonstrated in Fig. 2 [37].

According to the CFA which examined the degree to which patterns of means and covariation mirrored the conceptual model of parent activation, the data seem to fit a theoretical two-factor model of parent activation. The two-factor model appears as a merged version of the former theoretical four-factor model, with Factor 1 comprising the items 1 to 7, covering the concepts ‘beliefs’, and ‘confidence and knowledge’, and Factor 2 comprising the items 8 to 13, covering the concepts ‘action and perseverance’. The strong positive correlation (*r* = 0.72) between the factors suggests that the theoretical factors reflect the construct of parent activation as intuitively compiled into an inner cognitive factor and an outer behavioral factor, which are related.

#### Relevance to clinical practice

The literature has shown that patient activation may play a central role in improving mental health outcomes, including participation and treatment satisfaction [46]. The measurement of a generic and not disease-specific dimension of parent activation may be essential in the evaluation of parental health and the treatment efficacy for their child as well as in mental health research. Recent studies have shown that ADHD in children often affect parent–child interactions negatively and that parents of children with ADHD present with higher levels of stress and irrational beliefs related to emotion-focused strategies [47, 48]. Compared to other paediatric studies by Pennarola, DeCamp, Liberman and Yu [12, 14–16], we found higher activation scores for parents than what have previously been reported. The authors from a study of parent activation in a sample of caregivers of children in an emergency department [15] reported a mean activation score of 73 (*SD* = 15.74), while Yu et al. [16] reported a mean activation score of 67.36 (*SD* = 5.82) in a sample of caregivers of children with autism spectrum disorders. Even though the literature shows that parents of children with ADHD are presumed to experience high levels of burden and stress [49, 50], most parents in this study showed the highest level of activation with a mean score of 76.2. In the context that the parents responded to the P-PAM before a parental educational ADHD-specific course, the high activation level may indicate that the parents were engaged and proactive in managing their child’s health. There are several aspects that may explain the parents’ level of activation. By accepting the invitation to attend a parental educational course, which they had planned and signed up for in advance, the parents included in this study appeared to be engaged and proactive. Another aspect is the achievement of activation as a developmental process [4, 12]. Moreover, the parents’ developmental histories and personalities may influence how their parenting is sensitively attuned to their child’s capability, including emotional security, behavioural independence, social competence, and intellectual achievement [51].

### Strengths and limitations

One of the main strengths of this study is that it is the first to validate the Norwegian version of the P-PAM in an adequate sample of parents of children newly diagnosed with ADHD. With the inclusion of parents from four CAMHs, the quality of the study sample strengthens the generalisability of the results to other settings and populations. We also compare and discuss the importance of using different internal consistency coefficients such as Cronbach’s *α* and McDonald’s *ω* which may be of methodological value for future research. The questionnaire achieved good psychometric properties related to internal consistency and reliability, even though it is not in accordance with the intention of achieving four activation factors.

The limitations of the study must be considered. The cross-sectional design of the study prevented us from assessing the test–retest reliability and responsiveness. A methodological challenge also appeared when item 5 saturated nearly the same in the two factors of the scale, which might have caused a shift in the factors. However, excluding the item did not influence the main results, and was retained in the model. Moreover, the P-PAM could beneficially be explored and compared with a sample of parents of healthy children, which should be a subject for future studies. Due to strict ethical restrictions, we were not allowed to collect information, such as parental ADHD or other demographic or health-related variables, which may have provided valuable knowledge of the association with the activation levels [23, 52]. Furthermore, we did not collect information regarding paediatric health outcomes with the aim of evaluating the association between P-PAM scores and child health outcomes, although there is some evidence in the literature that shows that parental empowerment and engagement may improve child health outcomes [53]. Another limitation may be a possible influence of social desirability which is the tendency of the respondents to underreport socially undesirable attitudes and behaviours and to report more desirable attributes, which may indicate inaccurate self-reports and inconsistent study conclusions [54]. Indeed, several studies have highlighted the critical importance of confident, self-efficacious parents who actively manage their child’s health, and parental activation on behalf of their child; however, an analogous construct to patient activation in chronically ill adults has still not been fully illuminated [12].

## Conclusions

This study provides promising findings regarding the theoretical structure of parent activation as two related factors, involving ‘beliefs’ and ‘confidence and knowledge’ as an inner cognitive factor, and ‘action and perseverance’ as an outer behavioural factor. The model fit indices suggest an acceptable two-factor model, with high internal consistency and reliability between the factors. In addition, to evaluate and understand parent activation and how parent activation relates to the health outcomes of children with ADHD, further paediatric studies with the use of the P-PAM to call its psychometric properties into question are encouraged.

## Data Availability

The dataset used and analysed during the current study is available from the corresponding author on reasonable request.

## Abbreviations

ADHD: Attention Deficit Hyperactivity Disorder
CAMHS: Child and Adolescents Mental Health Services
CFI: Comparative Fit Index
CI: Confidence Interval
CFA: Confirmatory Factor Analysis
EFA: Exploratory Factor analysis
KMO: Kaiser-Maier-Olkin
PAM-13: Patient Activation Measure
P-PAM: Parent-Patient Activation Measure
RMSEA: Root Mean Square Residual
SD: Standard deviation
SE: Standard Error
SRMR: Standardized Root Mean Square Residual
TLI: Tucker Lewis Index
